# Rapid urban malaria appraisal (RUMA) I: Epidemiology of urban malaria in Ouagadougou

**DOI:** 10.1186/1475-2875-4-43

**Published:** 2005-09-16

**Authors:** Shr-Jie Wang, Christian Lengeler, Thomas A Smith, Penelope Vounatsou, Diallo A Diadie, Xavier Pritroipa, Natalie Convelbo, Mathieu Kientga, Marcel Tanner

**Affiliations:** 1 Swiss Tropical Institute (STI), P.O. Box, CH-4002 Basel, Switzerland; 2 Centre National de Recherche et de Formation sur le Paludisme, Ouagadougou (CNRFP), 01 B.P. 2208, Ouagadougou 01, Burkina Faso; 3 Ecole Inter-Etats d'Ingénieurs de l'Equipement Rural (EIER), 03 B.P. 7023, Ouagadougou 03, Burkina Faso

## Abstract

**Background:**

Rapid urbanization in sub-Saharan Africa has a major impact on malaria epidemiology. While much is known about malaria in rural areas in Burkina Faso, the urban situation is less well understood.

**Methods:**

An assessment of urban malaria was carried out in Ouagadougou in November -December, 2002 during which a rapid urban malaria appraisal (RUMA) was applied.

**Results:**

The school parasitaemia prevalence was relatively high (48.3%) at the cold and dry season 2002. Routine malaria statistics indicated that seasonality of malaria transmission was marked. In the health facilities, the number of clinical cases diminished quickly at the start of the cold and dry season and the prevalence of parasitaemia detected in febrile and non-febrile cases was 21.1% and 22.0%, respectively. The health facilities were likely to overestimate the malaria incidence and the age-specific fractions of malaria-attributable fevers were low (0–0.13). Peak prevalence tended to occur in older children (aged 6–15 years). Mapping of *Anopheles *sp. breeding sites indicated a gradient of endemicity between the urban centre and the periphery of Ouagadougou. A remarkable link was found between urban agriculture activities, seasonal availability of water supply and the occurrence of malaria infections in this semi-arid area. The study also demonstrated that the usage of insecticide-treated nets and the education level of family caretakers played a key role in reducing malaria infection rates.

**Conclusion:**

These findings show that determining local endemicity and the rate of clinical malaria cases are urgently required in order to target control activities and avoid over-treatment with antimalarials. The case management needs to be tailored to the level of the prevailing endemicity.

## Background

An estimated 200 millions people live in urban malaria endemic areas in Africa [1] and a high proportion of clinical admissions in these areas are treated as malaria. Urban malaria poses a major challenge for health care systems in Africa. The impact of urbanization and uncontrolled population growth on malaria endemicity needs to be established [2-5].

Epidemiological profiles and clinical patterns are known to vary between urban, suburban and rural environments in Burkina Faso [6]. Sabatinelli *et al*. [7]collected blood samples from 2,117 children aged 0–5 years in Ouagadougou and reported prevalence rates of 3.0%, 9.5%, 20.0%, 15.6%, 21.8% and 26.4% in sectors 1, 8, 11, 14, 22 and 23, respectively. These findings showed a gradient of malaria incidence from the centre (lowest risk) to the areas close to an artificial lake (highest prevalence). Dabire [8] further categorized the residence of patients into 1) town centre, 2) the areas across the canals and 3) the shore areas of the artificial lake or dam (*barrage*), reporting prevalence rates of 13.1%, 25.3% and 31.4%, respectively.

Entomological research in Ouagadougou dates back to the colonial period. The main vectors *Anopheles gambiae s.l., Anopheles funestus *and *Anopheles nili *were already identified in Ouagadougou by Le Gac *et al*., in 1945 [9]. Later, a longitudinal entomological survey [10,11] reported six species of *Anopheles *sp. mosquitoes with *An. gambiae s.l*. and *An. funestus *playing a key role during the dry season. The focality of malaria transmission was also noted. Concerns were also addressed about the breeding of *Anopheles *sp. vectors in large water reservoirs and in the water supply resources [12].

A standard study protocol Rapid Urban Malaria Appraisal (RUMA) was developed in June 2002 based on a WHO proposal and an Environmental Health Project draft protocol [13,14]. RUMAs were commissioned by Roll Back Malaria (RBM) for three francophone countries (Ivory Coast, Burkina Faso and Benin) and one anglophone country (Tanzania). Each of the four assessment reports provides the following: an overview of the urbanization history, an estimate of the fractions of malaria-attributable fevers, parasite rates for different areas, an outline of health care services and highlights of the "lessons learned" from the survey. A separate overview introduces this work in a wider framework [15].

The study aimed to compile a minimum dataset on urban malaria features in Ouagadougou within a period of six to ten weeks and to display the malaria risk in relation to population settlements, social and health care services, as well as the environment. The study aimed to provide essential information to better plan malaria control interventions.

## Methods

### Study sites and sample selection

Ouagadougou is the capital of Burkina Faso, situated between latitude 12.0 N-13.0 N and longitude 1.15 E-1.40 E, 300 meters above sea level. To the north, the vegetation thins out into sand dunes as it approaches the Sahara. The total area of Ouagadougou was around 570–655 km^2 ^in 2000 [16]. The annual precipitation is 750 to 900 mm. The rainy season is between June and October, the cold and dry season is between November and January and the hot and dry season is between February and May. The average temperature is approximately 19°C in January and 40°C from April to May. The total population in Ouagadougou was around 1,040,000 inhabitants in 2002 [17,18].

**Figure 1:**
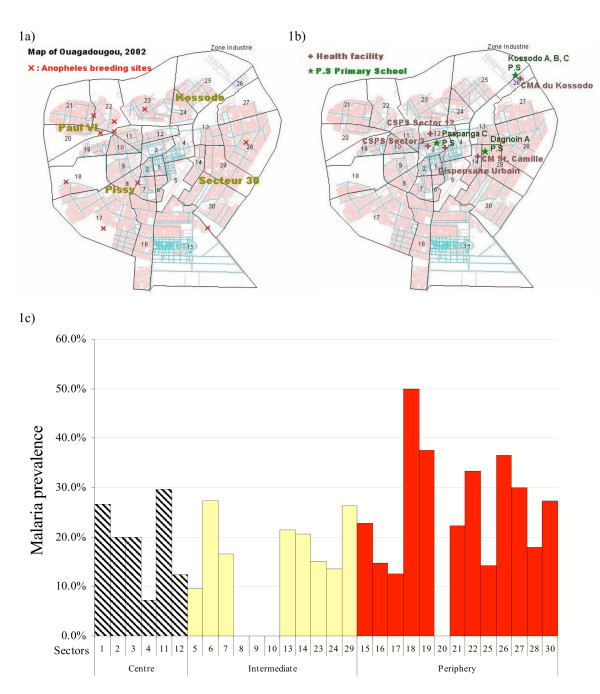
a) Map of sanitary districts and sectors, and *Anopheles *sp. breeding sites. Numbers indicate sectors. b) Map of selected sites for schools and health facility-based surveys. c) Malaria prevalence by sectors of Ouagadougou. Health facilities surveys.

The sanitary administrative structure is not identical to the political administrative structure. There are five administrative districts, which are divided into 30 urban sectors and 17 peripheral villages (Figure 1a). The four sanitary districts are Pissy (sectors 1–12 and 16–19) Kossodo (sectors 13, 23–27), Paul VI (sectors 20–22) and Secteur 30 (sectors 14, 15 and 28–30). Ouagadougou were classified into three different areas (centre, intermediate and periphery), according to their population size, distance to the centre and physical characteristics. The patterns of development and settlement, i.e. commercial, industrial areas, residential areas and natural environments (lakeside, forest or dry areas), were considered. It is a rapid assessment with limited budget; therefore, in each area only one health facility and school were selected for the surveys. Health facilities with a higher volume of outpatients per day were considered for the survey.

Centre: Due to low attendance in the urban dispensary (Dispensaire Urbain) which was originally selected, two sites were added to speed-up recruitment: Centre de Santé et de Promotion Sociale (CSPS) Paspange sector 12 and CSPS Dapoya sector 3 (Figure 1b). The selected dispensaries and the school, Paspanga C primary school (Figure 1b), are situated between the main dam and a busy commercial centre.

Intermediate areas: Dagnoin A primary school is situated in a poor residential area of sector 29, east of Ouagadougou (Figure 1b). An irrigation canal passes through this area. The missionary hospital St. Camille serves as a government Centre Médical avec Antenne Chirurgicale (CMA), with the highest attendance of patients in town.

Periphery: Kossodo A, B and C primary schools are situated in sector 26 at the north-east border behind the small forest Bois de Boulogne, which is a large farming area near an industrial zone. Centre Médical (CM) Kossodo is just opposite to the schools (Figure 1b). It is the only CM in Kossodo and it serves the majority of the population there.

To maximise case detection, the Centre National de Recherche et de Formation sur le Paludisme, Ouagadougou (CNRFP), situated in a busy area of the sector 4, near the University of Ouagadougou and CHN-YO, was identified as an additional site for the health facility-based fever survey (Figure 1b). It is not a health centre or clinic, but a national laboratory known for receiving self-referred malaria patients.

### RUMA Methodology

#### Review of literature and collection of health statistics

The author surveyed the articles published on urban malaria and the relevant thesis presented in the medical libraries in Ouagadougou. Demographic, health information and routine malaria reports were collected from the CNRFP, the Institut National de la Statistique et de la Démographie (INSD) and the statistics unit of the municipal health department.

#### Mapping of breeding sites

A rapid entomological survey was conducted with the assistance of CNRFP entomologists and produced a map of *Anopheles *breeding sites. At the beginning of the rainy season in 2002, there had been an entomological survey in Ouagadougou conducted by CNRFP, to identify mosquito breeding sites. In December, 2002, the entomological team double-checked all water bodies which were identified earlier. Due to time limitations, additional potential breeding sites were not checked. All larvae were collected and transferred to water containers. The containers were filled with the water taken from the breeding sites and were labelled with their location. These were delivered to the entomological laboratory of the CNRFP for hatching. The mature adult mosquitoes were then identified to species level. Geographic coordinates were recorded for all confirmed breeding sites of *Anopheles *sp. by Global Positioning System (GPS) (Garmin_ eTrex 12 canal GPS). A vector layer of a digital map of Ouagadougou with the locations of all health facilities and schools was provided by the GIS unit of the Ecole Inter-Etats d'Ingénieurs de l'Equipement Rural (EIER).

#### School parasitaemia surveys

School parasitaemia surveys were carried out to estimate endemicity in different transmission areas (high, medium and low). The school surveys were carried out from November 21 to 30, 2002, at the cold and dry season. Each school was close to the health facility chosen for the fever survey (see below) and was visited by the survey team (sociology students-interviewers, nurses, laboratory technicians and a field assistant). A series of meetings was held with teachers and schoolmasters to explain the purpose and methodology of the survey. Participation was voluntary and parents had to fill out a questionnaire and sign the consent form (See Additional file 1). Only children who returned the consent forms had a blood sample taken and axillary temperature measured. Samples were collected from 200 students aged six to ten years in each school. Children were interviewed with the assistance of schoolteachers regarding their socio-economic situation and malaria infection histories. Both thin and thick blood films were taken on the same slide and stained by Giemsa stain. Based on the assumption that 8000 white blood cells are found in one ml of blood, parasite density in thick smears was defined as the number of parasites per 200 white blood cells. If 200 white blood cells were identified and less than 9 malarial parasites found, the process was continued until 500 white blood cells were identified.

#### Health facility fever surveys

This methodology aimed to assess malaria prevalence in fever cases and estimate the age-specific fractions of malaria-attributable fevers and improve the case definition and clinical diagnosis of malaria. In urban areas, an estimated 5 % to 50% of fever cases among children under 15 years old were due to malaria. A sample size of 200 in each facility gave an estimate of the proportion of cases with parasites with the following approximate lower 95% confidence limits: for 5%, lower 95% CI: 2; for 50%, lower 95% CI: 46. The survey activities lasted for 18 days from December 1^st^, 2002. Over this period, 200 fever cases and 200 non-fever controls in each health facility were interviewed (See Additional file 2). About 50% of the sample was aged ≤ 5 years. Outpatients with a history of fever (past 36 hours) or with a measured temperature of ≥ 37.5°C were defined as cases. Controls were recruited from another department of the same clinic without current or past fever, and matched by age and residency. Infants with congenital abnormalities, patients with signs of severe disease, patients returning to the health facility for follow-up visits and patients who were not permanent town residents for more than six months per year were excluded from the survey. After being recruited and giving informed consent, each patient had an axillary temperature measurement and had a blood film taken. The odds ratio (OR) is the proportion of odds of having parasitaemia in fever cases over controls. The formula for the fraction of fever episodes attributable to malaria parasites is: (1-1/Odds Ratio)*P with P being the proportion of fever episodes in which the subjects also had malaria parasites present.

For quality control, 200 slides were re-examined by a senior technician of the CNRFP and then a second time at the Swiss Tropical Institute (STI). The sensitivity, specificity and accuracy rates of readings were 98.7%, 98.2% and 98.6%, which was considered excellent.

#### Brief description of the health care system

The diversity of malaria fever management has a critical impact on the early diagnosis and treatment of malaria. Planning malaria control in urban areas cannot be achieved without an overview of the distribution, coordination and practices of existing treatment providers. This evaluation involved a meeting with representatives of the CNRFP, EIER and the municipal health department. The senior officers facilitated the exchange of information, in particular through lists and maps of public and private health care providers.

### Statistical methods

The data were double-entered and validated in EpiInfo 6.04 (CDC Atlanta, USA, 2001). Data analysis was carried out in Stata 8 (Stata Corp. Texas, USA, 2003). Explanatory variables were age group, sex, residence, education level, axillary temperature, fever duration, bednet usage, urban agricultural activity, water resources and previous malaria infection within one month, as well as whether the patients had visited a rural area. The X^2 ^test was applied to assess associations between categorical variables. Logistic regression was performed to assess the association between binary outcomes and explanatory variables, adjusted for the confounding effects of age groups.

### Ethics

The Ethics Committee of the Ministry of Health of Burkina Faso and the research commission of the STI gave approval for the protocol. All the patients gave informed consent. In school surveys, the consent forms and questionnaires were delivered to the parents there days before the survey. Chloroquine (CQ) or amodiaquine (AQ) were paid if the patients presented fever signs.

## Results

From 1945 to 2002, 39 papers regarding urban malaria epidemiology in Ouagadougou were published in international journals. The documentation of urban malaria research in Burkina Faso is well organised; most research work was published in thesis of resident doctors reporting and analyzing data from their clinical practices. Previous studies showed that the malaria peak prevalence occurred in September and October (30%), while 80–95% of malaria cases are reported during the rainy season (July to mid-October) and up to six weeks afterwards. The reports of clinical malaria dropped from December onwards to reach the lowest point (3%) in June [8,19]. The overall parasite index was 16% in Ouagadougou in August and September, 1984 [7]. From January to December, 1988, 6,109 outpatients in three health facilities were examined and found that malaria infections were the main reason for fever episodes with a prevalence rate of 33% [20].

### Brief description of the health care system

**Figure 2:**
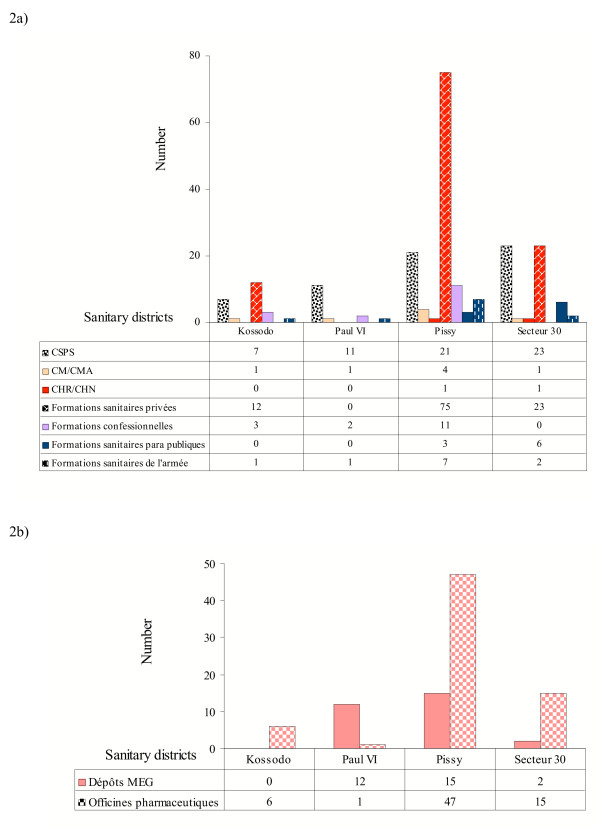
a) Categories and distribution of health services in Ouagadougou in 2002. Centre Hospitalier National (CHN)-National hospital. Centre Hospitalier Régional (CHR)-Regional hospital. Centre Médical (CM)-Health centre. Centre Médical avec Antenne Chirurgicale (CMA)-Health centre with an operating theatre. Centre de Santé et de Promotion Sociale (CSPS)-Dispensary and reproductive health unit. b) Distribution of pharmacies in Ouagadougou 2002. Dépôts MEG: Drug outlets. Officines pharmaceutiques: Prescription pharmacies.

The health care system in Burkina Faso is structured in five hierarchical levels (health post, health centre and district, regional and national hospitals). The public, private and voluntary services in Ouagadougou were heterogeneously distributed by district (Figure 2a). There were 80 public health facilities, 110 private health services (formations sanitaires privées) and 16 religious health facilities (formations confessionnelles). A total of 69 prescription pharmacies (officines pharmaceutiques) and 29 non-prescription drug outlets (Dépôts MEG) for essential medicines were registered in Ouagadougou in 2002 (Figure 2b). Over 60% of the prescription pharmacies were located in Pissy district (centre and south of city). Very few pharmacies were open in the intermediate and periphery areas. There was one dispensary (Centre de Santé et de Promotion Sociale) serving 23,800 inhabitants in Kossodo, 9,100 in Paul VI, 23,100 in Pissy and 12,400 in Secteur 30, respectively. There was no private health facility in Paul VI. This shows that the workload of public health services was heavy. Most of the inhabitants (76.0%, 83.4%, 72.4% and 64.2% in Kossodo, Paul VI, Pissy and Secteur 30, respectively) lived within 0–4 km of a public health facility, which means that the accessibility of services was good.

Although the policy of privatization of healthcare services was launched more than 15 years ago, the private sector in Ouagadougou has not developed as much as in other SSA cities. However, many private paramedical practices and drug outlets are not authorized and registered and, therefore, the number of private health facilities was certainly underestimated. Over a third of all health facilities were owned by the government in 2002.

### Results of malaria routine reports

**Figure 3:**
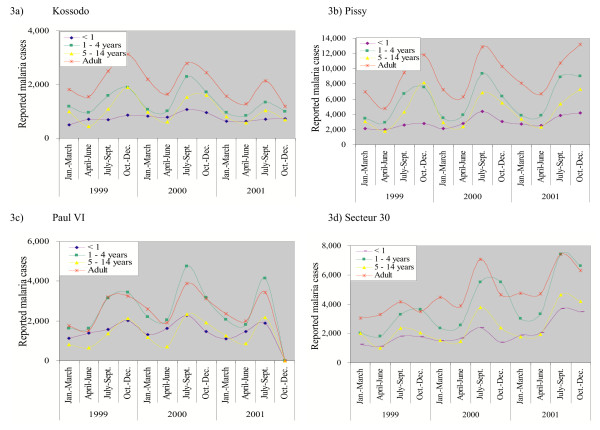
Reported simple malaria cases in Ouagadougou, by sanitary district, 1999–2002. Adult ≥ 15 years. a) Kossodo b) Pissy c) Paul VI d) Secteur 30

**Figure 4:**
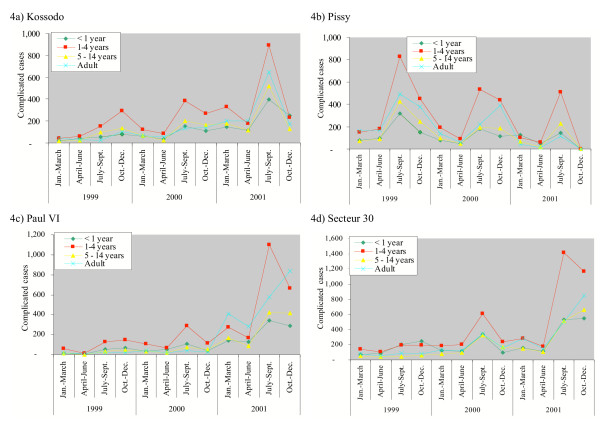
Reported complicated malaria cases in Ouagadougou, by sanitary district, 1999–2002. Adult ≥ 15 years. a) Kossodo b) Pissy c) Paul VI d) Secteur 30

Malaria morbidity and mortality data are reported from each health facility on a seasonal basis. The seasonal reports were available for 1999–2001, but not for 2002. The datasets were missing for the sanitary district Paul VI from October to December, 2001. The annual and seasonal patterns of mild and severe clinical malaria were marked (Figures 3 and 4). The highest incidence rates were reported from July to September and incidence rates went down from October to December. The lowest point was during the cold and dry season, from January to March. There was no big year-to year variation of reporting for simple malaria cases, while there was a sharp increase in reported complicated malaria during the rainy season in 2001. There was also a marked increase in reported cases in Pissy during the 1999 rainy season, for which no clear explanation could be found (Figure 4b).

In 2001, there were 203,466 mild malaria cases (30–40% of all consultations) reported among 596,365 consultations in the public health facilities of Ouagadougou (Table 1). There were 20,071 complicated malaria cases reported, which accounted for 1.9–4.9% of total consultations in the different age groups and 10% of all malaria cases. The original seasonal malaria reports were classified by age and gender using the following categories: one year old, one to four years old, five to 15 years old, male and female from 15 years-old. For infants and children under five years, one third of clinical consultations were due to malaria and one third was due to diarrhoea and lower respiratory tract infections. For children aged 5–14 years, malaria (41.4%), skin problems and wounds (14.2%), as well as lower respiratory tract infections (9.9%) were the major causes of consultations.

**Table 1:** Reported malaria cases and top 3 major causes for clinical consultations in Ouagadougou 2001.

Age category	Simple malaria	Severe malaria	LRTI¥	Skin/wounds	Diarrhoea	Total consultations
Infants < 1 year	31,430 (32.4%)	3,478 (3.6%)	15,348 (15.8%)	7,765 (8.0%)	16,306 (16.8%)	97,001
Children 1–4 years	58,070 (37.7%)	7,542 (4.9%)	20,592 (13.4%)	12,410 (8.0%)	20,338 (13.2%)	154,196
Children 5–14 years	38,283 (41.4%)	3,765 (4.1%)	9,120 (9.9%)	13,167 (14.2%)	3,424 (3.7%)	92,436
Adults ≥ years	75,683 (29.9%)	5,286 (2.1%)	20,862 (8.3%)	30,095 (11.9%)	10,884 (4.3%)	252,732
**Total**	**203,466 (34.1%)**	**20,071 (3.4%)**	**65,922 (11.1%)**	**63,437 (10.6%)**	**50,952 (8.5%)**	**596,365**

^¥^LTRI: Low Tract Respiratory Infection

### School parasitaemia surveys

**Figure 5:**
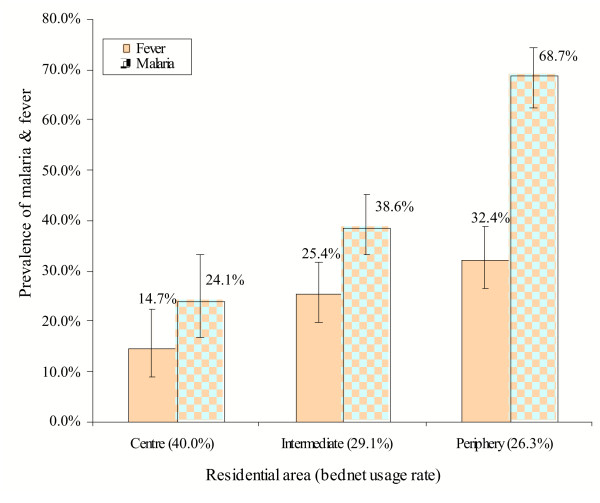
Malaria and fever prevalence in school children by area of residency. School parasitaemia survey. Vertical bars represent 95%CI.

*Plasmodium falciparum *and *Plasmodium malariae *were detected. The presence of a malaria infection was found in 285 out of 590 valid samples (48.3 %, 95% CI: 44.2–52.4). The prevalence of parasitaemia was 31.6%, 37.6% and 73.1% in Paspanga C, Dognoin A and Kossodo A, B, C primary schools, respectively. Each school had its own catchments area, although the children attending the same school lived in different districts of Ouagadougou. The children's residence was further classified; the prevalence rates were 24.1%, 38.6% and 68.7% in the centre, intermediate and periphery areas, respectively (Figure 5).

### Health facility-based surveys

Overall, 123/437 fever cases (22.0%) and 110/436 (20.1%) of controls were positive. The majority of infections were due to *P. falciparum*; very few cases of *P. malariae *and *Plasmodium vivax *were identified. The difference in parasitaemia rates between facility-based surveys and school surveys was likely to be due to the different age distribution of the sample populations: the mean age was 7.7 years in the school parasitaemia surveys and 19.9 years in the facility-based fever surveys.

In all four cities where RUMA was used, the age groups were classified using following categories: one year old, one to five years old, six to 15 years old and adults from 15 years-old. Table 2 shows that in the above age groups the parasites rates in febrile episodes were 12.1%, 25.9%, 37.1% and 18.0%, respectively, while 14.3%. 14.4%, 34.5% and 19.8 % of controls were parasitaemic. The six to 15 year old group of children was at the highest risk of malaria infection compared to infants and adults. Age-specific differences were only significant in the fever group (Table 2). The OR of having malaria in fever cases varied from 0.82 to 2.07 for different age groups. The estimated fractions of malaria-attributable fevers were low: -0.03, 0.13, 0.04 and -0.02 in the above age groups, respectively. A separate paper will give an overview of the fraction of fever attributed to malaria.

#### Gradients of malaria prevalence and breeding site mapping

**Figure 6:**
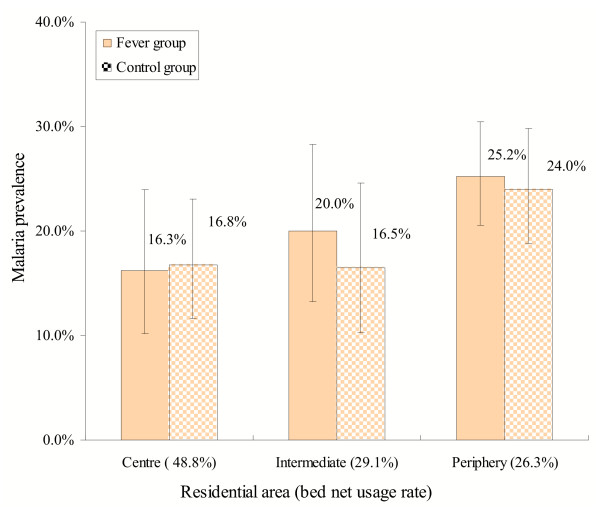
Malaria prevalence in fever cases and control groups by residential areas of patients. Health facility-based surveys. Vertical bars represent 95% CI.

The study population was categorized by their residence rather than the location of health facilities. Malaria infections were nearly equally found in both febrile episodes and afebrile controls in the study (Figure 6). Overall, the centre and intermediate areas of Ouagadougou were at a lower risk of malaria (prevalence: 14.1% and 20.7%) compared to the periphery of Ouagadougou (25.9%). Higher malaria infections were found among the residents from the periphery sectors 18 and 26 (Figure 1c). There was no malaria infection among the residents of sectors 8–10, where the government and business centres are located. The parasite rates in peripheral sectors such as 15–17 and 20 were surprisingly low, but this could be due to a small sample population.

In total, eight sites at the periphery and two sites in the centre were identified where *Anopheles *sp. larvae production was ongoing (Figure 1a). In the city centre, one *Anopheles *sp. breeding site was found in sector 8 where the canal passes and one in puddles/pools around artificial lake No. 2 in sector 11. In the periphery, the major open water bodies with productive *Anopheles *breeding were found in sectors 18, 19, 20, 21, 23, 28 and 30 (Figure 1a). The small temporary breeding sites in household compounds were not checked.

#### Socio-economic factors

The socio-economic status of study population was heterogeneous: over 70% of those in the city centre and around 50% of those in the intermediate and peripheral areas had at least a primary school level education. The proportion of households with tap water supply in the centre was higher (47.0%) than in the periphery (31.9%). Over 50% of the population relied on public fountains for water supply. There was no big difference in housing materials in the different areas (53.0–63.6% of houses were built with concrete and bricks and 3–5% were built with mud). An average of 42.2% of study population used a bednet the night before survey. The residents of the centre (48.8%) were more likely to sleep under a bednet than those in the intermediate (36.2%) and periphery areas (38.6%). This difference was significant (intermediate areas: OR = 0.59, 95% CI = 0.42–0.84, P < 0.001, periphery: OR = 0.66, 95% CI = 0.50–0.87, P < 0.001). More ITNs were present in households in the city centre (10.4%) than in the intermediate (7.5%) and the periphery areas (7.1%).

A logistic regression model was performed to estimate the association between socio-economic factors and malaria infection, adjusted for the effects of residential areas and age groups (Table 3). The risk of malaria was significantly reduced for people sleeping under a bednet (OR = 0.74, 95% CI = 0.54–1.00, P < 0.05). Neither education levels of the caretakers nor housing material were identified as significant risk factors. Having an urban agricultural land/garden near living compounds was positively associated with a malaria infection (OR = 1.39, 95% CI = 1.01–1.92, P < 0.05). Increased risk of malaria was associated with exposure to open water bodies, like fountains (OR = 1.66, 95% CI = 1.19–2.31, P < 0.005) or streams (OR = 2.80, 95% CI = 1.30–6.04, P < 0.005). Travelling to rural areas within 90 days was not correlated with the presence of parasitaemia.518 (47.5%) patients were treated for malaria within one month prior to the survey: 62.0% were treated at health centres and hospitals, while 35.0 % were self-treated at home or underwent no treatment. Very few people reported purchasing drugs in a pharmacy (2.2%) or using traditional medicine (0.8%).

**Table 2:** Odds ratio (OR) of having parasitaemia by age groups and fever/control groups. Health facility-based surveys.

Malaria prevalence	Fever	Controls	Fever			Controls		
Age groups			OR	95% CI	P value	OR	95% CI	P value

Infants 0–1 year	7/58 (12.1%)	3/21 (14.3%)	1	-	-	1	-	-
Children 1–5 years	45/174 (25.9%)	15/104 (14.4%)	2.54	1.08–6.00	<0.05	1.01	0.27–3.86	0.987
Children 6–15 years	23/62 (37.1%)	20/58 (34.5%)	4.30	1.67–11.03	<0.005	3.16	0.83–12.02	0.092
Adults >15 years	48/266 (18.0%)	72/363 (19.8%)	1.60	0.69–3.75	0.276	1.48	0.43–5.18	0.535

**Table 3:** Socio-economic factors and the risk of malaria infection by logistic regression model. Health facility-based surveys.

**Socio-economic factors**	**%**	**OR**	**95% CI**	**P value**
*Adjusted for the effects of residential areas and age groups*				

**Education**				
Primary	23.2%	1	-	-
Secondary	33.4%	0.97	0.62–1.49	0.873
Superior	5.2%	0.96	0.44–2.09	0.911
No education	35.5%	1.3	0.85–1.98	0.222
Religious	2.6%	0.74	0.24–2.27	0.594

**Housing material**				
Concrete/brick	58.1%	1	-	-
Leaf/mud	4.6%	1.61	0.82–3.19	0.17
Leaf	0.8%	2.13	0.50–9.00	0.304
**Others**	36.5%	**1.45**	**1.06–1.98**	**< 0.05**

**Water supply resource**				
Tap water	38.1%	1	-	-
Well	0.6%	1.58	0.18–13.90	0.68
**Fountain**	58.1%	**1.66**	**1.19–2.31**	**< 0.005**
**Others**	3.2%	**2.8**	**1.30–6.04**	**< 0.005**

**Living near a garden or agriculture land**				
No	71.0%	1	-	-
**Yes**	29.0%	**1.39**	**1.01–1.92**	**< 0.05**

*Adjusted for the effects of different residential areas*				

**Bednet usage**				
No use	58.0%	1	-	-
**Used**	42.0%	**0.74**	**0.54–1.00**	**< 0.05**

*Without adjusting for residential areas and age groups*				

**Rural exposure within 90 days**				
No	91.3%	1	-	-
Yes	8.7%	1.14	0.70–1.90	0.6

**Previous malaria treatment within 30 days with the presence of parasitaemia**				
No	52.5%	1	-	-
Yes	47.5%	1.1	0.82–1.48	0.5

### Monitoring of parasite resistance to anti-malarial drugs

The evolution of drug resistance of malaria in Burkina Faso has been described in two urban areas, Ouagadougou and Bobo-Dioulasso and is summarized in Table 4[21,22].

**Table 4:** Susceptibility of *P. falciparum *to antimalarials in Burkina Faso.

**Year**	**Drugs tested**	**Study sites**	**Urban/Rural**	**Authors**	**Failure rate *In vivo***
1982–1986	CQ (*in vitro & in vivo*)	Koudougou	Urban	[36]	First case found
1988–1989	CQ	Koudougou Zaghtouli Dori Banfora Fada N'Gourma	Urban Rural Urban Rural Rural	[37]	25%
1988	CQ	Zaghtouli	Rural	[38]	18.7%
1989	CQ	Dapelgo	Rural	[38]	20.2%
1982–1991	CQ, SP, quinine, MP (*in vitro*)	Ouagadougou Bobo-Dioulasso	Urban	[39]	6–15.8 %
1990–1992	CQ, SP (*in vitro*), quinine, halofantrine hydrochloride MP,	Ouagadougou and its neighbouring villages	Urban Rural	[22, 40-42]	CQ & SP: 8.1–24.4% Others: 0%
1993	CQ	Ouagadougou	Urban	[43]	25%
1995–1996	CQ, quinine, MP (*in vitro*)	Bobo-Dioulasso	Urban	[44]	CQ: 19–20% M:2–9.6%
1992–1998	CQ, AQ, quinine, halofantrine MP	Ouagadougou	Urban	[45]	AQ:4.3% & 2.2 % in 1997 CQ:8.5% in 1992, CQ:20% in 1994 H: 7.9% in 1997 (*in vitro*) MP: 0% in 1997 (*in vitro*) Q: 0.9% in 1995 MP+H:7.6% in 1997
1999–2002	CQ & SP	Bobo-Dioulasso	Urban	[46]	CQ:18% SP: < 1%

AQ: Amodiaquine

CQ: Chloroquine

MP: Mefloquine

SP: Sulfadoxine/pyrimethamine

## Discussion and conclusion

### RUMA methodology

The RUMA methodology is a cross-sectional study and the results may be different at another time of the year, or even vary between years.

Valuable information was extracted from the existing scientific literature and from health statistics. However, the research highlighted the need to enhance the capacity of municipal health department in collecting, processing, disseminating and using information. Some information needed for the evaluation of the health care system, such as the map with public/private health service providers and the schools had been prepared earlier by the GIS unit of EIER. A malaria map of breeding sites was produced during the cold and dry season mainly due to the help of CNRFP. They conducted an entomological survey in Ouagadougou at the beginning of the rainy season in 2002. Without such help, mapping breeding sites would not be possible in the frame of a RUMA.

### Mis-diagnosis of malaria

One-third of all clinical consultations were diagnosed as malaria cases and many of them are likely to be mis-diagnosed. Fever is no longer an indicative sign for the diagnosis of malaria As a result, a review of clinical guideline for the management of fever episodes is necessary in order to reduce over-treatment.

A heterogeneity of clinical signs of severe malaria was observed between the paediatric wards of the main hospital, the Centre Hospitalier National Yalgado Ouédraogo (CHN-YO) and a rural district hospital near Ouagadougou during the rainy season [6]. In town, the age distribution and the clinical spectrum of severe malaria were related to the place of residence of the patients. While Sanou [23] demonstrated that malaria remained a major cause of childhood morbidity and mortality in the main hospital in Ouagadougou during the rainy season 1993–94, the mis-diagnosis of malaria was reported to be close to 34.7% [24]. Dabire in 1990 showed that 63.4% of malaria cases in CHN-YO defined on clinical criteria alone were not parasitaemic [8]. The severity and diversity of malaria symptoms reflect the diversity of local malaria endemicity, leading to difficulties for malaria diagnosis. Presumptive treatment of malaria based uniquely on the fever sign leads to high rates of over-diagnosis and over-treatment. Hence, it is important to introduce diagnosis tests, among which rapid tests are very promising. If physicians then exclude malaria they should check for other causes of fever and initiate an appropriate therapy. All the cases confirmed by the rapid diagnosis test should be treated as malaria since the transmission level is not high.

### Parasitaemia results

In the school surveys, the parasitaemia rates were always higher than the febrile episodes since many children have asymptomatic infection. It was also observed that the community prevalence remained high at the beginning of the cold and dry season, while clinical malaria cases diminished quickly in health facilities. The control group had an even higher prevalence of parasitaemia than the fever group. The difference between parasitaemia rates in active and passive case detection was expected. The health facilities received patients from various communities, while school children mostly came from the same areas. This indicated that malaria infections were clustered in certain areas and that there were different levels of malaria transmission in Ouagadougou. Another explanation could be that some feverish patients may have taken paracetamol or another anti-pyretic and hence might not necessarily have presented with fever when visiting the dispensary.

### Urban agriculture and *Anopheles *vector breeding sites

The risk of a malaria infection in Ouagadougou might be associated with seasonal agricultural activities. A Ouagadougou home gardening map produced by Cissé and Gerstl showed that a majority of home gardens were situated at the city's outskirts and along the shores of artificial lakes and canals [25-27]. The irrigated plots along the dam and the water channels create rural enclaves within the city. A study conducted near Ouagadougou described a positive association between irrigated farming, the malariometric and malaria morbidity among children below five years of age [28]. In the health facility-based surveys, it was found that the water supply types (fountains or streams) and the proximity to home gardening fields were associated with malaria infection. This association was noted as well in Uganda [29] and Dar es Salaam [30].

### Malaria clusters in Ouagadougou

The parasitaemia prevalence rates in schools varied widely from the city centre to the periphery. The heterogeneity of endemicity within a small distance between the urban centre and the periphery of Ouagadougou was confirmed. Different levels of malaria transmission were previously found to be related to the spatial and temporal distribution of *An. gambiae *larval breeding sites in Ouagadougou [11,31]. Rossi *et al*. and Petrarca *et al*. concluded that higher prevalence rates of malaria occurred in areas where larvae breeding sites were semi-permanent. The most significant semi-permanent breeding sites are along the artificial lake, particularly in sectors 19–23. This was consistent with the observations mentioned above: higher prevalence rates were found along artificial lakes and canals, which are associated with urban agricultural activities.

### Awareness and practices of malaria prevention

ITNs coverage was not high, possibly due to economic constraints and availability problems. Annual household expenses for malaria treatment were estimated at 38,398 CFA [24]. The average cost of malaria treatments was 4,929 CFA per person in 1992 [32]. The average hospitalisation costs for complicated malaria have risen up to 21,160 CFA in the paediatric ward of CHN-YO [33]. Because of such high cost, the patients reported taking self-medication before visiting health facilities (35%) or increasingly invested in preventing mosquito bites (42.2%). These results agreed with the findings of local researchers who found 28–36% [7], 30.2% [34] and 50% [19] self-medication.

Some suggestions can be made concerning in-depth research and interventions. Firstly, emphasis urgently needs to be put on the proper diagnosis of "malaria cases", including the use of rapid tests [35]. Secondly, Ouagadougou has a huge artificial lake for its water supply and irrigation. It would be important to explore the malaria impact of current hydro-agriculture systems and urban agriculture activities during the cold and dry season and to find ways to mitigate their malaria potential. Thirdly, the cost for malaria treatment is relatively high in Burkina Faso, which leads to a high proportion of self-medication. The pricing policy in public health facilities should be revised as it should be considered an obstacle to case management and malaria control goals.

## List of abbreviations

AQ Amodiaquine

CHN Centres Hospitalier National

CHN-YO Centre Hospitalier National Yalgado Ouédraogo (CHN-YO)

CHR Centre Hospitalier Régional

CM Centre Médical

CMA Centre Médical avec Antenne Chirurgicale

CNRFP Centre National de Recherche et de Formation sur la Paludisme, Burkina Faso

CSPS Centre de Santé et de Promotion Sociale

CQ Chloroquine

INSD Institut National de la Statistique et de la Démographie

EIER Ecole Inter-Etats d'Ingénieurs et de l'Equipement Rural, Burkina Faso

GPS Global Positioning System

GIS Geographic Information System

ITNs Insecticide-Treated Nets

MP Mefloquine

OR Odds Ratio

RUMA Rapid Urban Malaria Appraisal

SP Sulfadoxine/pyrimethamine

STI Swiss Tropical Institute

## Authors' contributions

SW participated in the design of the study, conducted the field work, analysed and interpreted data, drafted and revised the manuscript. CL conceived the study, coordinated the field work and revised the manuscript. TS and PV participated in the design and statistical analysis. DD, XP and ES managed and supervised the data collection and laboratory work in the field. MK supervised the production of the breeding site map and the cleaning of this dataset. MT participated in the conception of the work, revised it critically at different stages and gave final approval of the version to be published.

## Note

Table 2. Odds ratio (OR) of having parasitaemia by age groups and fever/control groups. Health facility-based surveys.

## Supplementary Material

Additional File 1 School survey questionnaire: the questionnaire for school parasitaemia survey. Click here for file

Additional File 2 Hospital survey questionnaire: the questionnaire for health facility-based survey. Click here for file

## References

[B1] Keiser J, Utzinger J, Caldas de Castro M, Smith TA, Tanner M, Singer BH (2004). Urbanization in sub-saharan Africa and implication for malaria control. Am J Trop Med Hyg.

[B2] Donnelly MJ, McCall PJ, Lengeler C, Bates I, D'Alessandro U, Barnish G, Konradsen F, Klinkenberg E, Townson H, Trape JF, Hastings IM, Mutero C (2005). Malaria and urbanization in sub-Saharan Africa. Malar J.

[B3] Omumbo JA, Guerra CA, Hay SI, Snow RW (2005). The influence of urbanisation on measures of *Plasmodium falciparum *infection prevalence in East Africa. Acta Trop.

[B4] Hay SI, Guerra CA, Tatem AJ, Atkinson PM, Snow RW (2005). Urbanization, malaria transmission and disease burden in Africa. Nat Rev Microbiol.

[B5] Robert V, Macintyre K, Keating J, Trape JF, Duchemin JB, Warren M, Beier JC (2003). Malaria transmission in urban sub-Saharan Africa. Am J Trop Med Hyg.

[B6] Modiano D, Sirima BS, Sawadogo A, Sanou I, Pare J, Konate A, Pagnoni F (1999). Severe malaria in Burkina Faso: urban and rural environment. Parassitologia.

[B7] Sabatinelli G, Bosman A, Lamizana L, Rossi P (1986). Prevalence of malaria in Ouagadougou and the surrounding rural environment during the period of maximal transmission. Parassitologia.

[B8] Dabire E (1990). Morbidité et mortalité palustres au sein de la pathologie fébrile dans le service de pédiatrie de l'hôpital Yalgado Ouédraogo. MSc thesis.

[B9] Le Gac P, Seite P, Combescot de Marsaguet G (1945). Etude sur le paludisme à Ouagadougou. Bulletin de Extique la Societe de Pathologie Extique.

[B10] Bosman A, Sabatinelli G, Lamizana L (1988). Further observations on chemoprophylaxis and prevalence of malaria using questionnaire data in urban and rural areas of Burkina Faso. Parassitologia.

[B11] Rossi P, Belli A, Mancini L, Sabatinelli G (1986). A longitudinal entomologic survey on the transmission of malaria in Ouagadougou (Burkina Faso). Parassitologia.

[B12] Parent G, Ouedraogo A, Zagre NM, Compaore I, Kambire R, Poda JN (1997). Large dams, health and nutrition in Africa: beyond the controversy. Sante.

[B13] Warren M, Billing P, Bendahmane D, Wijeyaratne P (1999). Malaria in urban and peri-urban areas in sub-Sahara Africa. Environmental Health Project, activity report No.71.

[B14] WHO (2001). Rapid Urban Malaria Appraisal.

[B15] Wang S-J, Lengeler C, Smith TA, Vounatsou P, Cisse G, Diallo DA, Akogbeto M, Mtasiwa D, Teklehaimanot A, Tanner M Rapid urban malaria appraisal (RUMA) in sub-Saharan Africa. Malar J.

[B16] Institut National de la Statistique et de la Démographie (INSD) (2000). L'enquête Démographique et de Santé du Burkina Faso (EDSBF-II).

[B17] Municipale de Ouagadougou (2004). Commune de Ouagadougou. http://www.mairie-ouaga.bf/default.htm..

[B18] World Bank (2002). Upgrading of low income settlements, country assessment report, Burkina Faso.

[B19] Coulibaly SO (1989). La part du paludisme dans les affections fébriles en milieu urbain de Ouagadougou. Etude menée dans trois dispensaires de la ville.

[B20] Coulibaly CO, Guiguemde TR, Lamizana L, Ouedraogo JB, Dabiret E (1991). The role of malaria in febrile diseases in the urban environment of Ouagadougou (Burkina Faso, West Africa). Ann Soc Belg Med Trop.

[B21] Aouba AE (1992). Analyse de la situation de la chimiorésistance du paludisme au Burkina Faso: conséquences thérapeutiques.

[B22] Del Nero L, Lamizana L, Nebie I, Pietra V (1993). Susceptibility of *Plasmodium falciparum *to chloroquine and mefloquine in Ouagadougou area (Burkina Faso). Parassitologia.

[B23] Sanou I, Pare J, Traore S, Modiano D, Kam KL, Kabore J, Lamizana L, Sawadogo SA, Guiguemde TR (1997). Clinical signs of severe malaria in a pediatric hospital in Ouagadougou. Santé.

[B24] Coulibaly N (1993). Etude de l'impact économique du paludisme et du niveau de l'immunité humorale des maladesen zone rurale de Bobo-Dioulasso (Burkina Faso).

[B25] Cissé G (1997). Impact sanitaire de l'utilisation d'eaux usés et polluées en agriculture urbaine: Cas du maraîchage à Ouagadougou (Burkina Faso). thèse de doctorat No 1639.

[B26] Cissé G, Kientga M, Ouédraogo B, Tanner M (2002). Développement du maraîchage autour des eaux de barrage à Ouagadougou: quels sont les risques sanitaires à prendre en compte?. Cahiers Agricultures.

[B27] Gerstl S (2001). The economic costs and impact of home gardening in Ouagadougou, Burkina Faso. PhD thesis.

[B28] Zamane H (2000). Indices paludométriques et morbidité palustre chez les enfants de 0 à 59 mois dans les hydroaménagements agricoles de la vallée du Sourou en 1999 (Burkina Faso).

[B29] Staedke SG, Nottingham EW, Cox J, Kamya MR, Rosenthal PJ, Dorsey G (2003). Short report: proximity to mosquito breeding sites as a risk factor for clinical malaria episodes in an urban cohort of Ugandan children. Am J Trop Med Hyg.

[B30] Caldas de Castro M, Yamagata Y, Mtasiwa D, Tanner M, Utzinger J, Keiser J, Singer BH (2004). Integrated urban malaria control: a case study in Dar es Salaam, Tanzania. Am J Trop Med Hyg.

[B31] Petrarca V, Petrangeli G, Rossi P, Sabatinelli G (1986). Antimalarial campaign program in Ouagadougou (Burkina Faso): the *Anopheles gambiae *complex in the city of Ouagadougou and surrounding villages. Ann Ist Super Sanita.

[B32] Dajoari M (1992). Contribution à l'évaluation de l'impact économique du paludisme chez les travailleurs d'entreprises: Etude menée à l'Office de santé des travailleurs de Bobo-Dioulasso.

[B33] Sanon VM (1999). Etude du coût financier direct de la prise en charge du paludisme grave en milieu pédiatrique de Ouagadougou.

[B34] Traore S (1994). Formes graves de paludisme au service de pédiatrie du centre hospitalier national Yalgado Ouédraogo: Aspects épidémiologiques et cliniques.

[B35] Othnigué N, Wyss K, Tanner Marcel, Genton Blaise Urban malaria in the Sahel: Prevalence and seasonality of presumptive malaria and parasitaemia at primary care level in Chad.

[B36] Baudon D, Devoucoux R, Roux J, Sondo B (1984). Sensitivity of *Plasmodium falciparum *to chloroquine in the hyperendemic malaria savannah zone of Burkina Faso ex-Upper Volta. Use of *in vivo *and *in vitro *tests. Demonstration of a resistant strain in vitro. Bull Soc Pathol Exot Filiales.

[B37] Pietra V, Lamizana L, del Nero L, Rotigliano G (1992). *In vivo *chemosensitivity of *Plasmodium falciparum *to chloroquine in Burkina Faso: development of resistance 1988–1990. Parassitologia.

[B38] Kabore B (1991). Surveillance de l'efficacité therapeutique de la chloroquine a Ouagadougou en 1989–1990.

[B39] Guiguemde TR, Aouba A, Ouedraogo JB, Lamizana L (1994). Ten-year surveillance of drug-resistant malaria in Burkina Faso (1982–1991). Am J Trop Med Hyg.

[B40] Del Nero L, Lamizana L, Pietra V, Rotigliano G (1993). A national survey of the prevalence of chloroquine resistant *Plasmodium falciparum *in Burkina Faso. J Trop Med Hyg.

[B41] Del Nero L, Lamizana L, Nebie I, Sare S, Bougouma L, Pietra V (1994). *In vivo *sensitivity of *Plasmodium falciparum *to halofantrine hydrochloride in Burkina Faso. Am J Trop Med Hyg.

[B42] Del Nero L, Nebie I, Soudouem G, Pietra V (1994). Chloroquine and sulfadoxine/pyrimethamine sensitivity in Burkina Faso. *In vivo *sensitivity of *Plasmodium falciparum *to chloroquine and sulfadoxine/pyrimethamine in Burkina Faso. Trop Geogr Med.

[B43] Nabalma S (1994). Relation paludisme grossesse chez la femme enceinte à Ouagadougou: Intérêt d'une méthode de surveillance de chloroquino-résistance de *P. falciparum *reposant sur l'apposition placentaire couplée au dosage de chloroquinémie.

[B44] Ouedraogo A (1998). Etude *in vivo *de l'activité antiplasmodique de l'extrait hydroalcoolique de Gardenia Sokotensis Hutch (Rubiaceae) chez la souris NMRI infestée par *Plasmodium bergheio *(Burkina Faso).

[B45] Djire AA (1999). Analyse de l'évolution de la chimiorésistance du paludisme du Burkina Faso de 1992–1998.

[B46] Tinto H, Zoungrana E, Coulibaly S, Ouedraogo J, Traore M, Guiguemde T, Van Marck E, D'Alessandro U (2002). Chloroquine and sulphadoxine-pyrimethamine efficacy for uncomplicated malaria treatment and haematological recovery in children in Bobo-Dioulasso, Burkina Faso during a 3-year period 1998–2000. Trop Med Int Health.

