# Supplementary material for: Rapid urban malaria appraisal (RUMA) I: Epidemiology of urban malaria in Ouagadougou
Source: Malar J. 2005 Sep 16;4:43. doi: 10.1186/1475-2875-4-43 (PMC1261532; doi:10.1186/1475-2875-4-43)
Supplement: Additional File 1 — School survey questionnaire: the questionnaire for school parasitaemia survey. [file 1475-2875-4-43-S1.doc]

**Examen de la parasitémie – étude transversale dans les écoles**

**Prélèvement sanguin**

1 Depuis quand êtes vous à Ouagadougou ? ________________ ans

2 Température corporelle ____ °C

3 Résultat Plasmodium : Négatif [0] ou positif [1] --------

(a) Espèce parasitaire : P falciparum Charge __________ pa/uL de sang

P vivax Charge __________ pa/uL de sang

P ovale Charge __________ pa/uL de sang

P malariae Charge __________ pa/uL de sang

4 Site de l’étude : __________________

5 Numero d’enqueté (1-800) : _______

6 Lieu d’habitation : ____________________________________ (vérifier sur la carte)

7 Age : _____ ans _______ mois

8 Sexe : Masculin [1] ou Feminin [2] ______________

**Histoire de voyage et de la maladie**

9 Avez-vous été dans une zone rural durant ces trois derniers mois passés ?

(a) Date___/____/_____ Lieu : ______________ Durée : __________

(b) Date___/____/_____ Lieu : ______________ Durée : __________

(c) Date___/____/_____ Lieu : ______________ Durée : __________

10 Lieu de naissance : Ouagadougou [1], autre ville [2], zone rurale [3], autre pays [4] _____

11 Avez-vous été traité pour le paludisme durant le mois écoulé ? oui [1] /non [2] ________ (si non Q14)

12 Ou avez-vous été traité ? Domicile [1], pharmacie [2], centre de santé [3], hôpital [4], herbes locales[5], dormir [6], guérisseur [7], autre [8] _____________

13 Si autre, préciser ______________________________________________________

14 Qui est le responsable de la santé à la maison ? Mère [1], père [2], sœur ou frère plus âgé [3], grand parents [4], autre membres de la famille [5], autre [6] ___________

15 Utilisation de moustiquaire durant la nuit passée : oui [1], non [0] ____________

16 Moustiquaire traité ? oui [1], non [0] ____________

17 Maison : ciment/briques cuites [1], paille/terre [2], paille [3], autre [4] ____________

18 Source d’eau : robinet [1], puits [2], fontaine publique [3], autre [4] __________________
