# Supplementary material for: Rapid urban malaria appraisal (RUMA) I: Epidemiology of urban malaria in Ouagadougou
Source: Malar J. 2005 Sep 16;4:43. doi: 10.1186/1475-2875-4-43 (PMC1261532; doi:10.1186/1475-2875-4-43)
Supplement: Additional File 2 — Hospital survey questionnaire: the questionnaire for health facility-based survey. [file 1475-2875-4-43-S2.doc]

**Etude détection passive de cas de fièvres palustres**

**Critères d’inclusion d’enquête**

1. Combien de temps le patient est déjà à Ouagadougou ? ___________ ans
2. Qu'avez-vous fait avant de venir ? rien [1], médicament à la maison [2], hôpital de référence ou autre centre de santé, [3] pour des visites de contrôle [4], herbes locales [5], Paracétamol [6], Guérisseur traditionnel [7], pharmacie [8], autre [9] ______
3. Si autre, précise__________________________________________________________________
4. Température corporelle ___ ___. ___ ^0^C
5. Combien de jours de fièvre avant de visiter le médecin ici? _______ jours
6. Contrôle [0] ou Cas de fièvre [1] :_____________
7. Numéro d’enquêté (1-450) : ___ -___- _________
8. Site de l’étude: Arrondissement:____, ________________________________
9. Lieu d’habitation: Quartier : ________, N°de Carré : ______________________ (vérifier sur la carte!)
10. Age : ___ ans ___ mois ou Date de naissance :___/___ /____
11. Sexe: Masculin [1] ou Féminin [2]  _________

**Données socio-économiques**

1. Niveau d’instruction du patient (ou mère pour enfant de < 5 ans) : Premiere [1], Secondaire [2], Supérieure [3], Non [4], Autre [5], ________________________________
2. Utilisation de moustiquaire durant la nuit passée: Oui [1] ou Non [0] _______
3. Moustiquaire traitée ?: Oui [1] ou Non [0] _______
4. Maison: ciment/briques cuites [1], paille/terre [2], paille [3], autre [4] _____
5. Source d’eau ? robinet [1], puit [2], fontaine publique [3], autre [4] ____
6. Avez vous un champs agricole chez vous ou aux alentours de votre maison? Oui [1] ou Non [0] ____

**Histoire de voyage et de maladie**

1. Avez-vous été dans une zone rurale durant les 3 mois passés? Oui [1], Non [0] ____
2. Date____/____/____Lieu :_______________________________________ Durée :___ semaines
3. Date____/____/____Lieu :_______________________________________ Durée :___ semaines
4. Date____/____/____Lieu :_______________________________________ Durée :___ semaines
5. Lieu de naissance: Ouagadougou [1], autre ville [2], zone rurale[3], autre pays [4] ________
6. Avez-vous été traité pour le paludisme durant le mois écoulé? Oui [1] / Non [2] _____ (si non, Q 23)
7. Où avez-vous été traité? Domicile [1], pharmacie [2], centre de santé [3], hôpital [4], herbes locales [5], laisser l'enfant dormir [6], guérisseur [7], autre [8] ____________
8. Si autre, préciser ___________________________________________________________________

**Diagnostic clinique**

1. Symptômes (choix multiple): fièvre [1], froid/frissons [2], diarrhée [3], vomissement [4], mal de tête [5], vertiges [6], blessure [7], mal de ventre [8], sang dans l'urine [9], démangeaison [10], autres [11] ________
2. Si autres, précise___________________________________________________________
3. Diagnostics présomptif: ______________________________________________________________

**Prévèlement sanguin**

1. Résultat Plasmodium: Négatif [0] ou positif [1] ________
2. Espèce parasitaire: Charge __________ p.a/µL de sang

|  | P. falciparum | P. Vivax | P. Ovale | P. malariae |
| --- | --- | --- | --- | --- |
| Trophozoite |  |  |  |  |
| Scehizoite |  |  |  |  |
| Gametocyte |  |  |  |  |
